# Efficient One-Pot Synthesis of TiO_2_/ZrO_2_/SiO_2_ Ternary Nanocomposites Using *Prunus × Yedoensis* Leaf Extract for Enhanced Photocatalytic Dye Degradation

**DOI:** 10.1155/2022/3088827

**Published:** 2022-09-09

**Authors:** Velu Manikandan, J. Saranya Packialakshmi, Bandna Bharti, Palaniyappan Jayanthi, Ranjithkumar Dhandapani, Palanivel Velmurugan, Duraisamy Elango, Ragul Paramasivam, Vinayagam Mohanavel, Asad Syed, Abdhallah M. Elgorban, Ali H. Bahkali, Saravanan Muthupandian

**Affiliations:** ^1^Department of BioNano Technology, Gachon University, 1342 Seongnam-daero, Sujeong-gu, Seongnam-si, Gyeonggi-do 13120, Republic of Korea; ^2^Department of Agricultural Microbiology, GKVK, University of Agricultural Sciences, Bangalore 560065, India; ^3^School of Civil and Environmental Engineering, Harbin Institute of Technology, Shenzhen 518055, China; ^4^Department of Environmental Science, Periyar University, Salem, 636011 Tamil Nadu, India; ^5^Research and Development Division, Chimertech Private Limited, Chennai, 600082 Tamil Nadu, India; ^6^Centre for Materials Engineering and Regenerative medicine, Bharath Institute of higher, Education and Research, Chennai, Tamil Nadu, India; ^7^Department of Botany and Microbiology, College of Science, King Saud University, P.O. 2455, Riyadh, Saudi Arabia; ^8^Division of Biomedical Sciences, College of Health Sciences, School of Medicine, Mekelle University, Ethiopia; ^9^AMR and Nanotherapeutics Laboratory, Department of Pharmacology, Saveetha Dental College, Saveetha Institute of Medical and Technical Sciences (SIMATS), Chennai, 600 077 Chennai, India

## Abstract

A simple, efficient, and ecofriendly method was employed to synthesize TiO_2_/ZrO_2_/SiO_2_ ternary nanocomposites using *Prunus × yedoensis* leaf extract (PYLE) that shows improved photocatalytic and antibacterial properties. The characterization of the obtained nanocomposites was done by X-ray powder diffraction (XRD), Fourier transform infrared spectroscopy (FTIR), Raman spectroscopy, field-emission scanning electron microscopy (FE-SEM), and energy-dispersive X-ray spectroscopic (EDS) analysis. The synthesized ternary nanocomposites with nanoscale pore diameters were investigated for the elimination of Reactive Red 120 (RR120) dye. The obtained results showed about 96.2% removal of RR120 dye from aqueous solution under sunlight irradiation. Furthermore, it shows promising antibacterial activity against *Staphylococcus aureus* and *Escherichia coli*. The improved photocatalytic and antibacterial activity of TiO_2_/ZrO_2_/SiO_2_ may bring unique insights into the production of ternary nanocomposites and their applications in the environment and biomedical field.

## 1. Introduction

The Reactive Red 120 is a cationic dye used in several industries such as paper, leather, cotton, pulp, and wool. This toxic aqueous solution causes various problems including human health issues such as abnormal heartbeat rate, shock, cyanosis, jaundice, tissue death, carcinogenesis, mutagenesis, teratogenesis, respiratory toxicity, kidney failure and malfunctioning, and damage to the major organs like the brain, lung, liver, and sexual organs and causes emphysema [1–3]. The effluents released from the dyeing units are highly toxic, and it is directly discharged into the water bodies, which severely impact the environment. Due to the effluent's resistance to light, heat, chemicals, and water, these effluents at low-level concentrations do not readily degrade in the environment [4]. The estimated annual production of dye products is 10 million kilograms, out of which up to 2 million kilograms of reactive dyes enter the biosphere [5]. However, over a long period, different methods are employed for treating dyes from aquatic media [6], which include catalytic oxidation, sonocatalytic degradation, ozonation, adsorption [7], electrochemical oxidation [8], and coagulation/flocculation [9]. But these technologies are not suitable to meet the required water quality standards [10]. Recently, semiconductor photocatalysis drags the attention because of its advanced and green technique that provides the potential to degrade the dye in wastewater. It also stands for environmental benignancy, stability, and safety [11].

The growing global research needs a fast and environment-friendly technology to remove wastewater pollutants. Recently, nanotechnology has helped researchers enable potential applications in environmental issues. Titanium dioxide (TiO_2_) nanoparticles have been used as a benchmark photocatalytic material due to their high stability, nontoxicity, low cost, and strong oxidizing agent [12–15]. They have been used in various environmental applications such as high photocatalytic efficiency, optical, dielectrics, and antimicrobial devices [16–18]. TiO_2_ has a unique series of reactive oxygen functional groups present on the surface, such as carboxylic acid, hydroxyl, and epoxide groups that are beneficial in preparing the TiO_2_ nanocomposites [19] such as titanium oxide-reduced graphene oxide (TiO_2_-rGO) [20], SiO_2_-TiO_2_/ZrO_2_ [21], and Zr-TiO_2_/SiO_2_ [22]. Overall, titanium reveals a more extensive phi-conjugation system in a two-dimensional planar structure, a higher surface area, and a higher electron conductivity, including reactive oxygen and a carboxyl group which is present in titanium dioxide as a unique functional group would support strengthening the metal and metal oxide particles [23]. Under UV light irradiation [24], TiO_2_/ZrO_2_/SiO_2_ photocatalyst had greater photocatalysis performance than the TiO_2_/SiO_2_ and TiO_2_/ZrO_2_ systems. Furthermore, TiO_2_ is a promising material for removing dye from wastewater because of its high electron mobility and flexibility [25].

Several investigations are currently being conducted on the heterogeneous photocatalytic degradation of dye exposed to UV-A or visible light [26]. The oxidative technique has decolorized and mineralized the variety of azo dyes on a workbench scale using artificial illumination and solar energy [27]. Wang et al. successfully demonstrated the photocatalytic degradation of eight commercial dyes by using solar energy, including MeO, in the TiO_2_ mixture [28]. The plant-mediated synthesis of ternary nanocomposites is a safe, ecofriendly, rapid, and more stable approach due to the presence of numerous bioactive compounds such as alkaloids, flavonoids, terpenoids, tannins, saccharides, phenol, vitamins, amino acids, proteins, and various enzymes [29, 30]. Antibiotic resistance of microorganisms poses a serious problem. Thousands of people succumb to the harmful microorganisms and die due to hospital pathogens being resistant to the antibiotics. Hence, there is an ultimate need for novel antimicrobials, and the synthesized ternary nanocomposites exhibit a biocompatible structure which provides great convenience for medical applications. These phytochemicals present in the PYLE extract act as a reducing agent in the bioreduction of ions for the synthesis of TiO_2_/ZrO_2_/SiO_2_ nanocomposites. Controlled pore size distribution, density, compressive strength, and other unique characteristics are important properties of nanocomposites synthesized using the one-pot method. Moreover, the use of solar irradiation, instead of UV light, is another attractive point that fits today's requests for more intense use of renewable energies. The present study deals with the photocatalytic degradation of RR120 dye obtained by the one-pot solar photoreduction (with the intensity of ~50000 1x) to avoid the high temperature and pressure required for the standard hydrothermal reaction. Additionally, the antibacterial activity of the green synthesized TiO_2_/ZrO_2_/SiO_2_ nanocomposites was also investigated.

## 2. Experimental Sections

### 2.1. Materials

Titanium tetraisopropoxide (C_12_H_28_O_4_Ti assay ≥ 98%, Sigma), zirconium (IV) acetylacetonate (Zr(C_5_H_7_O_2_)_4_ > 98%, Sigma), sodium silicate (Na_2_SiO_3_·9H_2_O), and methanol (Daejung Chemicals Reagents, South Korea) were used as precursors to prepare oxide composites. Reactive Red 120 was supplied by SD Fine Chemicals, Mumbai, India. Stock solutions of 1000 mg/L of the dye were prepared, and further concentrations were obtained by diluting the stock solution. Mueller Hinton Agar (MHA) was purchased from MB cell, South Korea. All solutions were prepared using Milli Q water with of conductivity 18 *μΩ*/m.

### 2.2. Biological Materials

*Staphylococcus aureus* and *Escherichia coli* were collected from the Korean agriculture culture collection (KACC) in Suwon, South Korea. The collected organisms were subcultured in nutrient agar and incubated at 37°C for 24 hours; after incubation, the plates were stored at 4°C until the experiment.

### 2.3. *Prunus × Yedoensis* Leaf Extracts (PYLE) Preparation

50 grams of *Prunus× yedoensis* leaves was collected from the plants in and around the Chonbuk National University, Iksan campus, South Korea. The collected leaves were washed in distilled water to remove the impurities, and then these leaves were sheared into small pieces. After shearing, it was boiled with 200 mL sterile Milli Q water for 30 min in a heating mantle to obtain the decoction. The obtained decoction was subjected to filtration using a Whatman No.42 filter paper. After filtration, it was stored at 4°C until further process.

### 2.4. One-Pot Green Synthesis and Calcination Treatment of TiO_2_-Coated ZrO_2_/SiO_2_ Nanocomposites

100 mL of 0.1 M titanium tetraisopropoxide (C_12_H_28_O_4_Ti assay ≥ 98%, Sigma) solution was added to 300 mL of *Prunus × yedoensis* leaf extract mixture was vigorously stirred for one hour and ultrasonicated for 15-30 min. Followed by adding 100 mL of 0.1 M Zr(C_5_H_7_O_2_)_4_ and 100 mL of 0.1 M Na_2_SiO_3_·9H_2_O to the sonicated reaction mixture using a peristaltic pump (flow rate-0.5 mL/hour) at 80°C. Then the reaction mixture was subjected to centrifugation at 12000 rpm, the pellets were collected, and then the pellets were washed subsequently with distilled water and methanol to remove organic material present in the final product. The obtained product was subjected to calcination at 800°C for two hours in a muffle furnace. The final product obtained was designated as TiO_2_/ZrO_2_/SiO_2_ ternary nanocomposites and subjected to characterization and investigation of photocatalytic and antibacterial activities.

### 2.5. Characterization of TiO_2_/ZrO_2_/SiO_2_ Ternary Nanocomposites

The green synthesized TiO_2_/ZrO_2_/SiO_2_ ternary nanocomposites were analyzed by FE-SEM-EDS (SU-8240, Hitachi, Japan) to observe the surface morphology and appearance of the nanocomposite. EDS was used to confirm the element present in the nanocomposite. XRD patterns were obtained using a Rigaku X-ray diffractometer to find out the crystalline nature of the nanocomposite. The scanning was completed in the region of 2*θ* from 10°-80° at 0.041/min at a constant time of 2*S*. The functional groups in the prepared nanocomposite were analyzed using a Perkin-Elmer FTIR spectrophotometer (USA) with a range of 4000-400 cm^−1^. Raman spectroscopy was carried out by XploRA Raman microscope equipped with laser light of wavelength 532 nm. The UV-visible spectroscopy was recorded on UV-1800, Shimadzu, Kyoto, Japan, with the variable wavelength from 300 to 800 nm.

### 2.6. Photocatalytic Activity of TiO_2_/ZrO_2_/SiO_2_ Ternary Nanocomposites under Various Light Sources

The photocatalytic activity of TiO_2_/ZrO_2_/SiO_2_ ternary nanocomposites was conducted by the method proposed by Borthakur et al. [31] with modifications. Briefly, the reaction mixture was prepared by adding 10 mg of synthesized TiO_2_/ZrO_2_/SiO_2_ ternary nanocomposites in 100 mL of RR120 aqueous solution with an initial concentration of 10 ppm, and the suspension was stirred in the dark for 30 min before irradiated in the sunlight to establish the absorption-desorption equilibrium between the photocatalyst and RR120 dye. The sunlight irradiation was carried out between 11:00 a.m. and 2:00 p.m. on consecutive days (summer season) in June and July 2020 (GPS coordination: 35.84682° N, 127.12935° E). The photocatalytic activity of TiO_2_/ZrO_2_/SiO_2_ ternary nanocomposites was estimated by measuring the degradation of RR120 in an aqueous solution under sunlight irradiation using a UV-Vis spectrophotometer. A sample solution of RR120 was withdrawn within the fixed time interval and centrifuged at 12,000 rpm for 20 min during the photocatalytic reaction. After centrifugation, the supernatant was used to measure the amount of RR120 at *λ*_max_ (515 nm) [26] using a UV-visible spectrophotometer. The following equation determined the percentage of RR120 degraded by the prepared nanocomposite from the photocatalytic activity [32].

(1) $$\begin{array}{c} Percentage\;\text{of }degradation=\frac{C_{0}-C_{t}}{C_{0}}\times 100. \end{array}$$

Here, *C*_0_ is the initial concentration of the aqueous RR120 solution (mg/L), and *C*_*t*_ is the concentration of the aqueous solution after irradiation (mg/L). The reaction mixture was also subjected to different pH (4–12), different initial concentrations (*C*_0_) of RR120 (10 mg/L to 30 mg/L), and three different dosages of TiO_2_/ZrO_2_/SiO_2_ (from 20 to 60 mg/L). The kinetics study of RR120 was carried out to find the reaction rate constant of as-synthesized TiO_2_/ZrO_2_/SiO_2_ nanocomposites. The degradation of RR120 can be described by the pseudo-first-order equation [33].

(2) $$\begin{array}{c} \;\ln\left(\frac{C_{0}}{C_{t}}\right)=-k_{app}t, \end{array}$$

where *C*_0_ is the initial concentration of RR120 aqueous solution, *C*_*t*_ is the final concentration of RR120 aqueous solution, *k*_app_ is the pseudo-first-order rate constant (min^−1^), and *t* is time (min). *k*_app_ values were enumerated from the slope ln(*C*_*t*_/*C*_0_) at a different time plot observed for the sample solution without a catalyst, with the catalyst in a dark chamber, TiO_2_/SiO_2_, TiO_2_/ZrO_2_, and TiO_2_/ZrO_2_/SiO_2_ photocatalysts. The *k*-rate constant was derived through the kinetic value.

### 2.7. Antibacterial Activity

The TiO_2_/ZrO_2_/SiO_2_ ternary nanocomposite antibacterial activity was evaluated against the *Staphylococcus aureus* and *Escherichia coli* by the well diffusion technique. The bacterial suspension was swabbed on the Muller Hinton agar plate for antibacterial activity, and the different concentrations of nanocomposites (50, 75, and 100 *μ*g/mL) were loaded in the respective well on the agar plates. Then the plates were incubated at 37°C for 24 h. The antibacterial activity of TiO_2_/ZrO_2_/SiO_2_ ternary nanocomposites was identified by the clear zone around the well, which indicates the inhibition of bacteria by the composite.

## 3. Results and Discussion

This study prepared the green synthesis of TiO_2_/ZrO_2_/SiO_2_ ternary nanocomposites using PYLE.

### 3.1. X-Ray Diffraction Analysis

**Figure 1:**
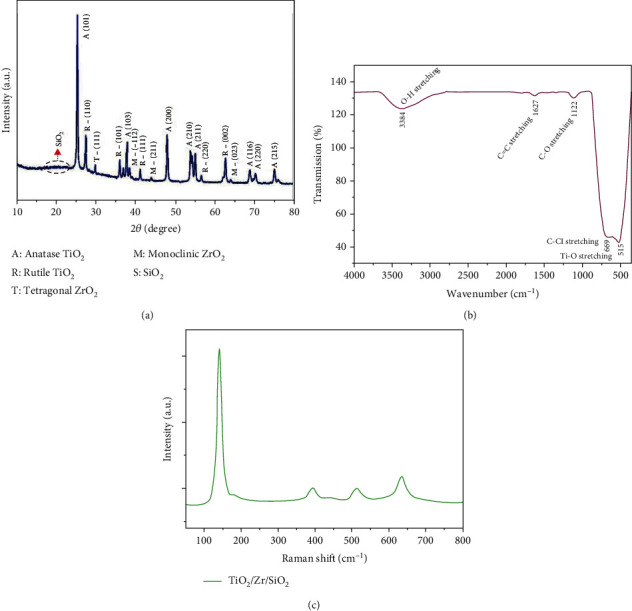
(a) XRD spectra, (b) FTIR spectrum, and (c) Raman spectra of TiO_2_/ZrO_2_/SiO_2_ nanocomposite.

Figure 1(a) illustrates the XRD pattern of TiO_2_/ZrO_2_/SiO_2_ nanocomposites. The diffraction peaks at 25°, 36°, 48°, 54°, 55.5°, 69°, 70°, and 75° were assigned to the corresponding planes (101), (103), (200), (210), (211), (116), (220), and (215), respectively, which indicate the presence of anatase TiO_2_. Also, it can be evidenced that the moderate low-intensity peaks at 27°, 35.5°, 42°, 56°, and 63° correspond to the planes of (110), (101), (111), (220), and (002), revealing the presence of rutile TiO_2_. Similar interpretations were made by Chellappa and Vijayalakshmi [34]. The prepared TiO_2_/ZrO_2_/SiO_2_ ternary nanocomposite might exhibit a mixed phase of anatase and rutile TiO_2_. A low intense peak at 30° indexed to (111) resembles the zirconia phase, which might increase the mechanical strength of the ternary nanocomposites. The diffraction peaks at 38°, 45°, and 65° correspond to (112), (211), and (023), respectively; these planes confirmed the presence of monoclinic ZrO_2_. This was in good agreement with the study conducted by [35]. The heterojunction of TiO_2_-coated ZrO_2_/SiO_2_ inhibits the conversion of TiO_2_ anatase to rutile at 800°C. In ZrO_2_/SiO_2_, the low intense peak at 20° and 30° reflects the combination pattern of ZrO_2_/SiO_2_ into TiO_2_ coated nanocomposite [36, 37]. In this study, all assigned diffraction peaks in TiO_2_-coated ZrO_2_/SiO_2_ revealed an enhanced photocatalytic activity compared with the pure TiO_2_ because of the anatase crystalline phase. From the XRD results, it can be assumed that the coated TiO_2_ nanoparticles might be entered into the channels of the ZrO_2_/SiO_2_ binary particle network.

### 3.2. FTIR Analysis

The FTIR spectrum of the nanocomposites is shown in Figure 1(b). Several functional groups were present in the prepared nanocomposite within 4000-400 cm^−1^. The prominent peak at 3384 cm^−1^ corresponds to the O-H stretching group present on the composite surface. The bank at 1627 cm^−1^ corresponds to C=C stretching vibrations in aromatic functional groups [38]. It is well known that the surface hydroxyl group plays a significant role in photocatalysis by capturing the holes and generating reactive hydroxyl radicals with high oxidation capacity [36]. The band around 1122 cm^−1^ is attributed to C-O stretching vibrations from the alcohol group, and the band at 669 cm^−1^ is assigned to the C-Cl stretching vibrations in the alkyl halide group. However, introducing titanium atoms to SiO_2_ to form the tetrahedral structure gives a complete structure of adsorbent [39]. The small peak at 515 cm^−1^ corresponds to the alkyl group in the Ti-O stretching, confirming the metal-oxygen bonding to nanocomposite [40].

### 3.3. Raman Analysis

Raman spectra of TiO_2_/ZrO_2_/SiO_2_ ternary nanocomposites are shown in Figure 1(c). The sharp peaks at 140, 394, 510, and 635 cm^−1^ were assigned to the anatase phase of TiO_2_. No characteristic peaks of silica were observed in the spectra. It is well known that zirconia has three polymorphs, namely monoclinic, tetragonal, and cubic; however, no Raman bands were observed for all these polymorphs [21]. Therefore, the Raman results indicate that silica and zirconium oxide are amorphous in the prepared TiO_2_/ZrO_2_/SiO_2_ ternary nanocomposites and are probably present as dispersed surface species.

### 3.4. FE-SEM-EDS

**Figure 2:**
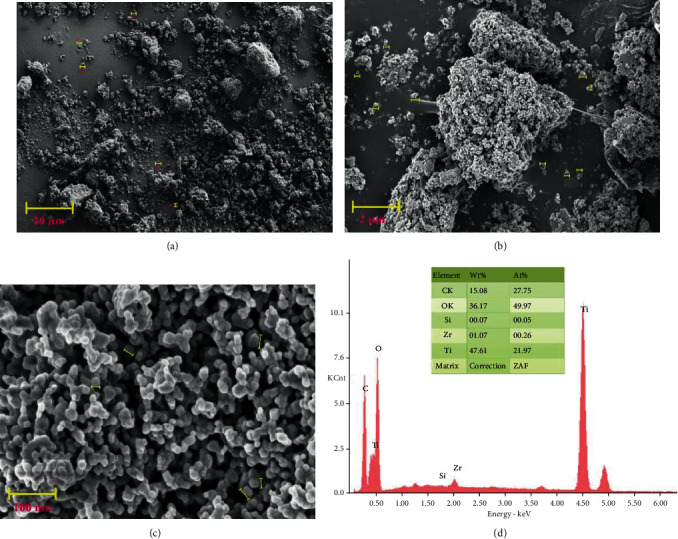
(a–c) FE-SEM images of TiO_2_/ZrO_2_/SiO_2_ ternary nanocomposites and (d) EDS spectra of TiO_2_/ZrO_2_/SiO_2_ ternary nanocomposites.

The surface morphology of TiO_2_/ZrO_2_/SiO_2_ nanocomposite was observed under field-emission scanning electron microscopy (FE-SEM). TiO_2_/ZrO_2_/SiO_2_ ternary nanocomposites were almost spherical in shape [41]. The uniform distribution of particles on the nanocomposite surface has been observed. The formation of aggregation in the nanocomposite might be due to the presence of excess H^+^ ions of H_2_O molecules on the surface of the nanocomposite [42]. The particle size of the prepared nanocomposite was in the range of 2-20 *μ*m, as shown in Figures 2(a)–2(c), where the prepared particles appeared to have a moderately rough surface [43]. Also, the surface roughness of nanocomposite was slightly increased due to the presence of TiO_2_ on the surface [44]. The elemental composition of TiO_2_/ZrO_2_/SiO_2_ ternary nanocomposites was determined by energy-dispersive X-ray spectroscopy (EDS), as shown in Figure 2(d). It can be seen that the presence of different elements such as titanium (Ti), zirconium (Zr), and silica (Si) confirmed the configuration of the green synthesized TiO_2_/ZrO_2_/SiO_2_ ternary nanocomposites. Biofabrication of nanocomposites was reduced and capped by polyphenols, which was actively confirmed by EDS [42].

### 3.5. Elemental Mapping-EDS Analysis

**Figure 3:**
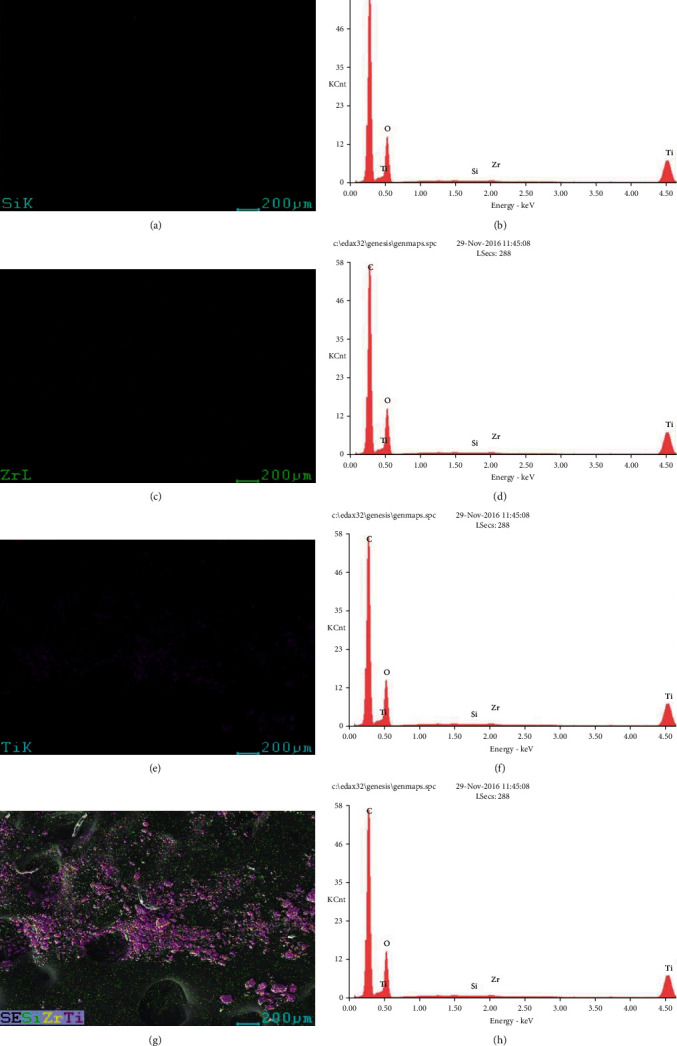
(a, c, e, g) Element mapping analysis of TiO_2_/ZrO_2_/SiO_2_ ternary nanocomposites and (b, d, f, h) EDS spectra.

Elemental mapping results of TiO_2_/ZrO_2_/SiO_2_ nanocomposites are shown in Figures 3(a), 3(c), 3(e), and 3(g). The EDS results assessed the homogeneity of the nanocomposites. The distribution of Si, Zr, and Ti in the TiO_2_/ZrO_2_/SiO_2_ nanocomposites is observed in Figures 3(b), 3(d), 3(f), and 3(h). Several earlier studies well coincided with the evenly distributed titanium over the SiO_2_ spheres, which evidently confirms the active role of titanium in the nanocomposites [45, 46]. EDS measurements confirmed the presence of Ti, Zr, and Si. Furthermore, all other elements like Zr, Si, and O are uniformly dispersed without agglomeration, and similar results were obtained from the study conducted by Choi and Choy [47].

### 3.6. Photocatalytic Degradation of RR120 in the Presence of TiO2/ZrO2/SiO2 Ternary Nanocomposites

**Figure 4:**
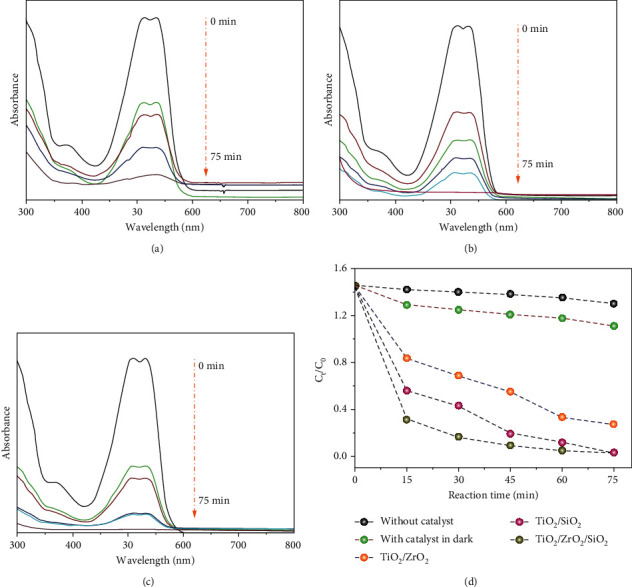
UV-Vis spectra of RR120 removal at different time intervals: (a) TiO_2_/ZrO_2_, (b) TiO_2_/SiO_2_, and (c) TiO_2_/ZrO_2_/SiO_2_ ternary nanocomposites and (d) RR120 removal rate at different photocatalytic systems.

The prepared TiO_2_/ZrO_2_, TiO_2_/SiO_2_, and TiO_2_/ZrO_2_/SiO_2_ nanocomposites were tested for their photocatalytic activity. The photocatalytic degradation of the RR120 dye experiment was standardized with the pH, catalyst dose, concentration of RR120 dye, and time interval under the sunlight irradiation. We have identified that at pH of 5, a catalyst dose of 10 mg/L, the concentration of RR120 dyes with 30 mg/L, and initially at 15 minutes, steadily increasing the steady time of 75 minutes showed better photocatalytic activity. The RR120 dye absorption peak at 515 nm showed that the amount of dye is drastically decreased without shifting the peak position to the baseline, indicating the complete decomposition in the aqueous solution. Moreover, these bioreduced TiO_2_/ZrO_2_/SiO_2_ nanocomposite*s* toward RR120 exhibited a promising photocatalytic efficiency with a 96.2% removal rate within 75 min. The absorbance spectra of the treated dye in the aqueous solution with the prepared composites were measured using a UV-visible spectrophotometer (Figures 4(a)–4(c)). The variations in the relative RR120 dye concentration (*C*_*t*_/*C*_0_) are presented in Figure 4(d), which shows the comparison of the photodegradation rate of RR120 dye in the presence of TiO_2_/ZrO_2_, TiO_2_/SiO_2_, and TiO_2_/ZrO_2_/SiO_2_ nanocomposites, respectively. The variation in RR120 dye removal is influenced by sunlight sources irradiation time [48].

The obtained results clearly show that the RR120 removal efficiency of aqueous solutions without catalyst, with the catalyst in the dark, TiO_2_/ZrO_2_, and TiO_2_/SiO_2_ were less than that of the heterogeneous TiO_2_/ZrO_2_/SiO_2_ ternary nanocomposites. The plant-based nanocomposite preparation is the most attractive approach for mass production at a minimal price that is safe for the ecosystem and human life. This nanocomposite might be an excellent potential photocatalyst for degrading pollutants utilizing sunlight [49].

The fact that the nanocomposites were synthesized by the sol-gel method could describe the photocatalytic improvement under natural sunlight with TiO_2_/ZrO_2_/SiO_2_ ternary nanocomposites. In this case, nanocomposites with a large specific area would include more active surface sites for adsorbing water molecules and forming aggressive •OH and HOO• radicals by capturing the photogenerated holes [50]. The photocatalytic interactions are fueled by these active free radicals, resulting in the degradation of various pollutants from the aqueous medium [51]. However, the larger surface area still enables the dye molecule diminishment on the TiO_2_/ZrO_2_/SiO_2_ photocatalyst surface. According to Abroushan et al. [52], the phosphate molecules are adsorbed on the surface of nanocomposite and generate electrons when exposed to sunlight [52]. In the present study, these electrons are attracted by the surface adsorbed O_2_ molecules, resulting in the formation of O_2_•– and HO• radicals, which have a higher chance of contacting RR120 dyes due to a quicker reaction speed, and the RR120 dye may be mineralized over time by superoxide radical ions [53]. As an outcome, the relatively smaller crystalline size of TiO_2_/ZrO_2_/SiO_2_ ternary nanocomposite is advantageous for reducing O_2_ and oxidation of H_2_O molecules by capturing the electron, and the hole pair enhances the photocatalytic performance when exposed to sunlight [54]. According to the results, TiO_2_/ZrO_2_/SiO_2_ ternary nanocomposites as active photocatalyst have an excellent photocatalytic potential to discharge the RR120 dye in the aqueous solution. The results obtained were compared with the other green synthesized nanocomposites, as shown in Table 1. From the results, the TiO_2_/ZrO_2_/SiO_2_ photocatalyst has a unique adsorption capability for RR120 in an aqueous solution. This enhancement could be attributed to a synergistic effect on a unique adsorption property and effective electron detachment at the photocatalyst functionality of nanocomposites. Moreover, no photocatalyst based on TiO_2_/ZrO_2_/SiO_2_ ternary nanocomposites for the breakdown of RR120 dye contaminant has been reported until now.

### 3.7. Kinetic Study of the TiO_2_/ZrO_2_/SiO_2_ Ternary Nanocomposite Catalysts

**Figure 5:**
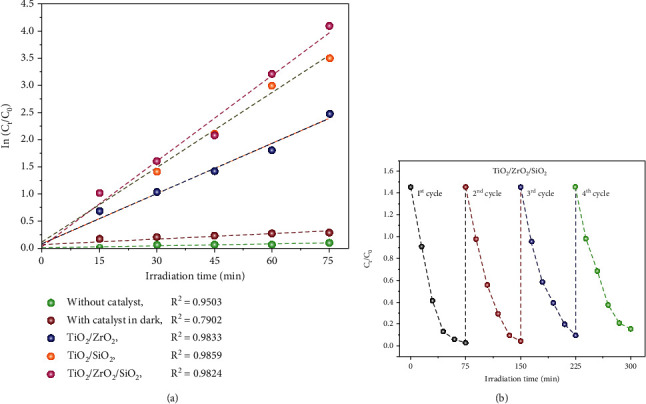
(a) Pseudo-first-order reaction kinetics of photodegradation of RR120 over various samples and (b) reusability of the prepared TiO_2_/ZrO_2_/SiO_2_ ternary nanocomposites.

Figure 5(a) illustrates the reaction kinetics of photodegradation of RR120 dye by various nanocomposites. The *k* value for a sample with catalyst in a dark chamber, without a catalyst, TiO_2_/ZrO_2_, TiO_2_/SiO_2_, and TiO_2_/ZrO_2_/SiO_2_ ternary nanocomposite was estimated to be *R*^2^ = 0.7902, 0.9503, 0.9833.0.9859, and 0.9824, respectively. Each of the plot lines shows a linear correlation with a high correlation coefficient of 0.9824, indicating the degradation of RR120 dye by green synthesized ternary nanocomposite using sunlight follows pseudo-first-order reaction kinetics. The higher *k* value proves that the TiO_2_/ZrO_2_/SiO_2_ ternary nanocomposite has an excellent photocatalytic activity. This statement was in agreement with the previous report conducted by Zhang et al. [55].

### 3.8. Reusability Study

For assessing the reusability of TiO_2_/ZrO_2_/SiO_2_ ternary nanocomposites, photodegradation was checked by repeating the four cycles as shown in Figure 5(b). The photocatalytic experiments (300 min) were repeated in four cycles, with the same nanocomposites retained after each cycle [56]. At the end of each photocatalytic experiment, samples were recovered and washed with deionized water and then used to analyze their stability. The amount of photocatalytic reduction by the nanocomposites showed the best performance, and similar interpretations were observed in the study conducted by Hinojosa–Reyes et al. [57]). These results depicted the photocatalytic activity of the biogenic fabricated TiO_2_/ZrO_2_/SiO_2_ ternary nanocomposites with renewed management. It can be considered that the distinction in the degradation efficiency might be due to the various surface features, crystalline nature, and the optical properties of nanocomposites [58]. After recycling four times, there were no significant variations in the degradation efficiency. This clearly states that the synthesized TiO_2_/ZrO_2_/SiO_2_ ternary nanocomposites are reusable enough for industrial and environmental applications.

### 3.9. Antibacterial Activity

**Figure 6:**
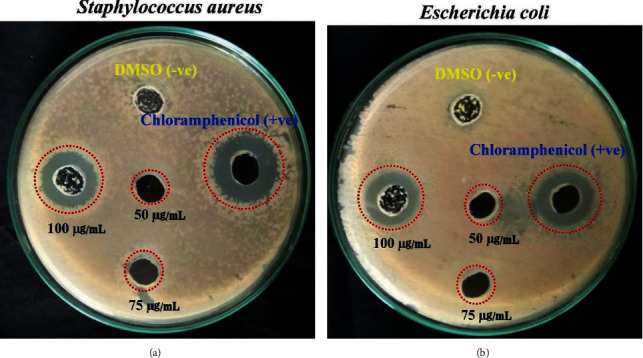
Variable numbers of colonies in agar plates (a) *Staphylococcus aureus* and (b) *Escherichia coli* exposed to different concentrations of TiO_2_/ZrO_2_/SiO_2_ nanocomposites.

The bactericidal activity of TiO_2_/ZrO_2_/SiO_2_ against *S. aureus* and *E. coli* is displayed in Figure 6. The antibacterial activity was carried out by a well diffusion method, and a zone of inhibition was observed for different concentrations (50, 75, and 100 *μ*g/mL) of a nanocomposite (20 mg in 0.5 mL) [59]. While increasing the concentration of the nanocomposites, the zone of inhibition in the well plate also increases. The highest inhibition rate was observed in *S. aureus* at the concentration of 100 *μ*g/mL, compared to *E. coli* (Table 2). The enhanced antibacterial activity of TiO_2_/ZrO_2_/SiO_2_ ternary nanocomposites has been familiar to the generation of reactive oxygen species (ROS), which could disrupt the DNA, proteins, and lipids, and leads to the death of the bacteria [60]. Nanoparticles can easily penetrate through the cell membrane; consequently, accumulation inside bacterial cells damages membrane integrity and ultimately destroys the bacteria [61]. While the nanocomposite was in contact with the cell membrane of the bacteria, Ti^2+^ was released and bound to the outer membrane; because the bacterial cell membrane was negatively charged [60]. Therefore, Ti^2+^ positively charged might mutually attract negatively charged cell membrane and cause damage to the cell membrane, which undoubtedly leads to bacterial death [62].

### 3.10. Photocatalytic Mechanism

**Figure 7:**
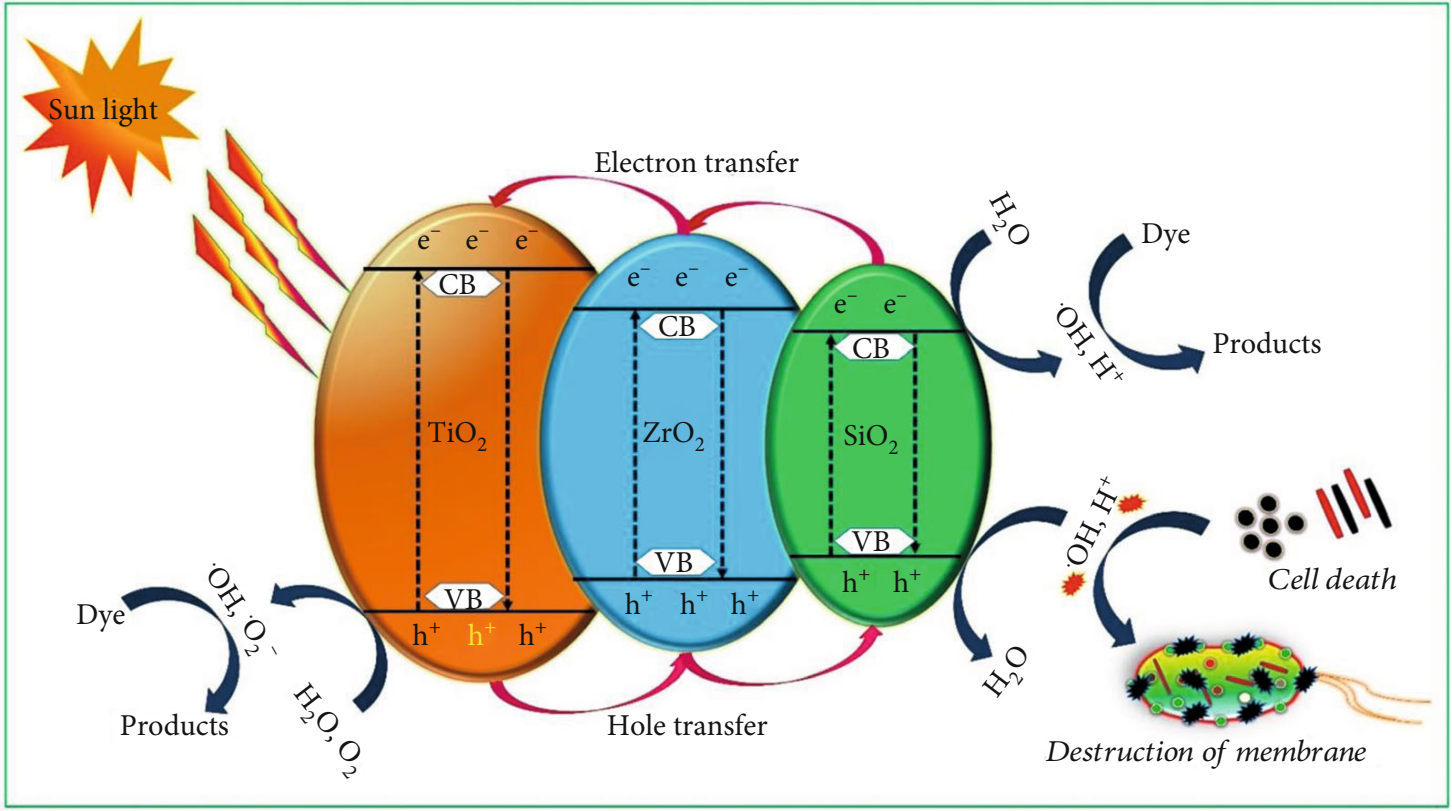
The proposed mechanism of RR120 degradation by TiO_2_/ZrO_2_/SiO_2_ ternary nanocomposites.

Figure 7 illustrates the proposed mechanism of RR120 degradation by TiO_2_/ZrO_2_/SiO_2_ nanocomposites. The photocatalytic process consists of the photogenerated electron and hole and their transfer to the surface of the nanocomposites and creates active oxygen species. Under the irradiation of artificial sunlight, each oxide's conduction and valence band contain the photogenerated hole and electron. According to the diagram, the position of the valence band of TiO_2_ is higher than that of ZrO_2_ and SiO_2_. Therefore, the photogenerated hole transfer can take place from the valance band of TiO_2_ to the valance band of ZrO_2_ and SiO_2_ [63]. However, in the case of photogenerated electrons, transfer takes place from the conduction band of SiO_2_ to ZrO_2_ and then to TiO_2_. Such photogeneration effectively reduces the recombination of electron-hole pairs. Hence, during the photocatalytic reaction, the electron can be captured by O_2_ to create a superoxide radical anion [55]. The hole can react with the hydroxyl group to generate hydroxyl radicals responsible for the degradation of RR120. It can be clear from the diagram that the hole and electron are effectively separated from each other at the surface of the TiO_2_/ZrO_2_/SiO_2_ nanocomposite.

## 4. Conclusions

TiO_2_/ZrO_2_/SiO_2_ ternary nanocomposites were successfully synthesized by a simple, efficient, and ecofriendly method. The obtained results showed that TiO_2_/ZrO_2_/SiO_2_ ternary nanocomposites have an excellent photodegradation activity and a promising bactericidal activity against the *Staphylococcus aureus* and *E. coli*. The high photocatalytic performance of TiO_2_/ZrO_2_/SiO_2_ was achieved based on the electron transfer between TiO_2_ NPs and ZrO_2_-SiO_2_. Also, the XRD pattern revealed the presence of monoclinic and tetragonal ZrO_2_ and anatase TiO_2_. The ternary nanocomposites decreased the recombination rate of electron and hole pairs; therefore, the superoxide anion, hydroxyl radical, and hydroxyl group play important role in the photodegrading and antibacterial activities. In nutshell, the one-pot green synthesized TiO_2_/ZrO_2_/SiO_2_ ternary nanocomposite act as a promising photocatalyst to remove the recalcitrant dye/pollutant in environmental applications and can be used for other industrial and medical applications.

## Figures and Tables

**Table 1:** Comparison of photocatalytic activity of TiO_2_/ZrO_2_/SiO_2_ nanocomposites with similar kinds of studies.

S. No	Catalyst	% removal	Catalyst (mg/L)	Irradiation time	References
1	Fe_2_O_3_/Ag nanocomposites	88.2%	60	140 min	[64] Saranya et al. [64]
2	TiO_2_ nanocomposites	94%	100	150 min	[65] Khade et al. [65]
3	TiO_2_–SiO_2_–Ag nanocomposites	80%	50	4 h	[66] Liu et al. [66]
4	Catechin@ZIF-L nanocomposites	92%	50 mg	150 min	[67] Raju et al. [67]
5	TiO_2_/ZrO_2_/SiO_2_ nanocomposites	96.2%	10 mg	75 min	Our study

**Table 2:** Antibacterial activity (zone of inhibition) of TiO_2_/ZrO_2_/SiO_2_ nanocomposites against Gram-positive and Gram-negative bacteria.

		Zone of inhibition (ZOI) in mm					
S. No	Samples	Bacterial strains					
		*S. aureus*			*E. coli*		
		50 *μ*g	75 *μ*g	100 *μ*g	50 *μ*g	75 *μ*g	100 *μ*g
1.	PYLE	—	—	—	—	—	—
2.	TiO_2_/ZrO_2_	—	1 ± 0.6	2 ± 0.2	—	1 ± 0.8	2 ± 0.4
3.	TiO_2_/SiO_2_	—	1 ± 0.2	1 ± 0.9	—	1 ± 0.5	2 ± 0.3
4.	TiO_2_/ZrO_2_/SiO_2_ nanocomposites	8 ± 0.6	11 ± 0.9	12 ± 0.9	6 ± 0.4	8 ± 0.3	10 ± 0.6
5.	Chloramphenicol	10 ± 0.7	12 ± 0.7	15 ± 0.3	12 ± 0.4	15 ± 0.3	16 ± 0.6

## Data Availability

All the data created were available within this manuscript.

## Abbreviations

PYLE: *Prunus × yedoensis* leaf extracts
KACC: Korean agriculture culture collection
ROS: Reactive oxygen species
XRD: X-ray powder diffraction
FTIR: Fourier transform infrared spectroscopy
FE-SEM: Field-emission scanning electron microscopy
EDS: Energy-dispersive X-ray spectroscopic
